# Genome-Wide DNA Methylation Profiles Indicate CD8+ T Cell Hypermethylation in Multiple Sclerosis

**DOI:** 10.1371/journal.pone.0117403

**Published:** 2015-03-03

**Authors:** Steffan D. Bos, Christian M. Page, Bettina K. Andreassen, Emon Elboudwarej, Marte W. Gustavsen, Farren Briggs, Hong Quach, Ingvild S. Leikfoss, Anja Bjølgerud, Tone Berge, Hanne F. Harbo, Lisa F. Barcellos

**Affiliations:** 1 Department of Neurology, Oslo University Hospital, Oslo, Norway; 2 Institute of Clinical Medicine, University of Oslo, Oslo, Norway; 3 Oslo Centre for Biostatistics and Epidemiology, Department of Biostatistics, University of Oslo, Oslo, Norway; 4 Epi-Gen, Institute of Clinical Medicine, Akershus University Hospital, University of Oslo, Oslo, Norway; 5 Genetic Epidemiology and Genomics Laboratory, Division of Epidemiology, School of Public Health, University of California, Berkeley, United States of America; Cleveland Clinic Foundation, UNITED STATES

## Abstract

**Objective:**

Determine whether MS-specific DNA methylation profiles can be identified in whole blood or purified immune cells from untreated MS patients.

**Methods:**

Whole blood, CD4+ and CD8+ T cell DNA from 16 female, treatment naïve MS patients and 14 matched controls was profiled using the HumanMethylation450K BeadChip. Genotype data were used to assess genetic homogeneity of our sample and to exclude potential SNP-induced DNA methylation measurement errors.

**Results:**

As expected, significant differences between CD4+ T cells, CD8+ T cells and whole blood DNA methylation profiles were observed, regardless of disease status. Strong evidence for hypermethylation of CD8+ T cell, but not CD4+ T cell or whole blood DNA in MS patients compared to controls was observed. Genome-wide significant individual CpG-site DNA methylation differences were not identified. Furthermore, significant differences in gene DNA methylation of 148 established MS-associated risk genes were not observed.

**Conclusion:**

While genome-wide significant DNA methylation differences were not detected for individual CpG-sites, strong evidence for DNA hypermethylation of CD8+ T cells for MS patients was observed, indicating a role for DNA methylation in MS. Further, our results suggest that large DNA methylation differences for CpG-sites tested here do not contribute to MS susceptibility. In particular, large DNA methylation differences for CpG-sites within 148 established MS candidate genes tested in our study cannot explain missing heritability. Larger studies of homogenous MS patients and matched controls are warranted to further elucidate the impact of CD8+ T cell and more subtle DNA methylation changes in MS development and pathogenesis.

## Introduction

Multiple sclerosis (MS) is a chronic, inflammatory disease of the central nervous system (CNS) and the leading cause of disability in the young Western population[1]. The knowledge of the underlying mechanisms is sparse, but points to a complex interplay between common genetic and environmental factors. Genome-wide association studies (GWAS) and earlier genetic studies have identified 110 MS-associated loci and alleles of the *HLA-DRB1* (most frequently *15:01) and *HLA-A* (*02) loci[2, 3]. Immunologically relevant genes, particularly those involved in T-helper cell differentiation, are significantly overrepresented among MS-associated variants[4]. Clinical and para-clinical evidence indicate MS results at least in part from inflammatory reactions in the CNS[5]. CD4+ T cells predominate in acute CNS lesions[6], whereas CD8+ T cells predominate in chronic lesions[7, 8], indicating an active role for these lymphocyte subclasses in MS.

Recently, epigenetic modifications have been shown to influence predisposition to complex diseases[9]. DNA methylation, the addition a methyl group to the cytosine in C-G dinucleotides (CpG-sites) modulates expression of nearby genes. DNA methylation associations have been reported for several autoimmune diseases, including Sjogren’s syndrome, systemic lupus erythematous and rheumatoid arthritis[10–12]. Investigation of genome-wide DNA methylation can be performed by the Infinium HumanMethylation450 BeadChip (450K)[13]. DNA methylation of different tissues is highly diverse and influenced by environmental factors, therapy or on-going disease processes[14]. Therefore, sample homogeneity is a requirement for successful investigations of the relationship between DNA methylation and phenotypes. However, in a clinical setting heterogeneous whole blood (WB) is easily accessible for MS patients, and whether disease relevant changes can be reliably detected in WB has not been determined.

DNA methylation studies of WB, or purified blood cells from MS patients have been performed for a small number of discordant twin pairs and siblings at genome-wide scale[15], or for candidate genes and a limited numbers of CpG-sites[16, 17]. Huynh *et al*. have shown that pathogen-free brain regions of MS patients have a different global and specific DNA methylation profile as compared to healthy donor brain samples[18]. More detailed DNA methylation profile studies in carefully characterized, homogenous MS samples are highly warranted. Here we present genome-wide DNA methylation results from purified CD4+ and CD8+ T cells and WB of female MS patients and healthy controls.

## Materials and Methods

### Samples and genotyping

A homogenous collection of 16 untreated, female Norwegian MS patients with relapsing remitting MS (RRMS) and 14 age-matched female controls were included (Table 1). All patients and controls were of self-declared Nordic ancestry. Patients were between ages 18 and 63 and recruited from the MS clinic at the Oslo University Hospital, Oslo, Norway. Controls were recruited either through the patients or among hospital employees. None of the patients had ever received immune-modulatory drugs. Patients had not experienced a relapse or received steroids in the three months prior to enrollment and fulfilled the updated McDonald MS criteria[19]. MRI of the CNS was performed within four weeks of blood sampling and the number of lesions and contrast-enhancing lesions was counted. The Extended Disability Status Scale (EDSS) was performed on the day of blood sampling.

**Table 1:** Characteristics of individual MS patients and summaries of patients and controls.

Patient	Age category^1^	Years MS^2^	EDSS^2^	MSSS^2^	OCB^3^	MRI lesions	Contrast lesions MRI^4^
1	3	11	3.50	4.13	Yes	>20	No
2	3	11	2.00	2.11	Yes	>20	No
3	6	33	2.50	1.14	No	>20	No
4	1	2	0.00	0.53	Yes	10–20	No
5	5	1	0.00	0.64	Yes	>20	No
6	2	8	1.50	1.90	Yes	>20	No
7	2	11	1.50	1.38	No	>20	No
8	4	6	5.00	7.61	Yes	10–20	Yes
9	5	11	0.00	0.17	Yes	>20	Yes
10	3	9	1.00	0.86	Yes	>20	No
11	2	6	2.00	3.51	Yes	>20	Yes
12	4	16	1.00	0.38	Yes	10–20	No
13	2	6	1.50	2.30	Yes	>20	Yes
14	5	3	2.50	5.98	Yes	10–20	No
15	2	6	2.00	3.51	Yes	10–20	No
16	4	1	1.00	2.34	Yes	10–20	No
Summarized							
Patients Mean (S.D.; range)	38.9(25–63)	8.8(7.7; 1–33)	1.7(1.3; 0–5)	2.4(2.1; 0.2–7.6)	14/16(87.5%)	N/A	4/16(25%)
Controls Mean (S.D.; range)	39.2(28–58)	N/A	N/A	N/A	N/A	N/A	N/A

^1^Age category: 1 = 25–29, 2 = 30–34, 3 = 35–39, 4 = 40–44, 5 = 45–49, 6 = 60–64.

^2^At inclusion in this study.

^3^Oligoclonal bands present in cerebrospinal fluid taken at time of diagnosis.

^4^Contrast enhancing lesions on MRI.

Abbreviations: EDSS = Expanded Disability Status Scale, MSSS = Multiple Sclerosis Severity Score, OCB = oligoclonal bands, MRI = Magnetic Resonance Imaging, S.D. standard deviation

Genome-wide single nucleotide polymorphism (SNP) genotypes for patients and controls were assessed using the Human Omni Express BeadChip (Illumina, San Diego, CA, USA). A large Norwegian GWAS dataset published earlier[20] was used to confirm Nordic ancestry of our MS patients and controls by principal component analysis (PCA) as implemented in the R(version3.0.3) software package[21] (S1A Fig.). Genotypes were imputed against the European 1000-genomes data using IMPUTE2[22]. Details on procedures are provided in S1 Materials and Methods.

### Ethics statement

The Regional Committee for Medical and Health Research Ethics South East, Norway, approved this study. Written informed consent was obtained from all study participants.

### DNA methylation profiling and data normalization

CD4+ and CD8+ T cells from WB were isolated for MS patients and controls in a semi-automated manner using the autoMACS Pro Separator (Miltenyi Biotec, Germany). DNA from WB and purified CD4+ and CD8+ T cell samples was extracted and treated with bisulphite. DNA methylation levels were assessed using the 450K (Illumina, USA). Raw data were exported from Illumina’s BeadStudio and normalized using the ‘BMIQ’ algorithm described previously[23]. Analyses were performed using beta values of methylation[24]. The CD4+ sample from donor 8 and both the CD8+ and WB sample from donor 3 had technical issues and were excluded before further analysis.

**Fig 1:**
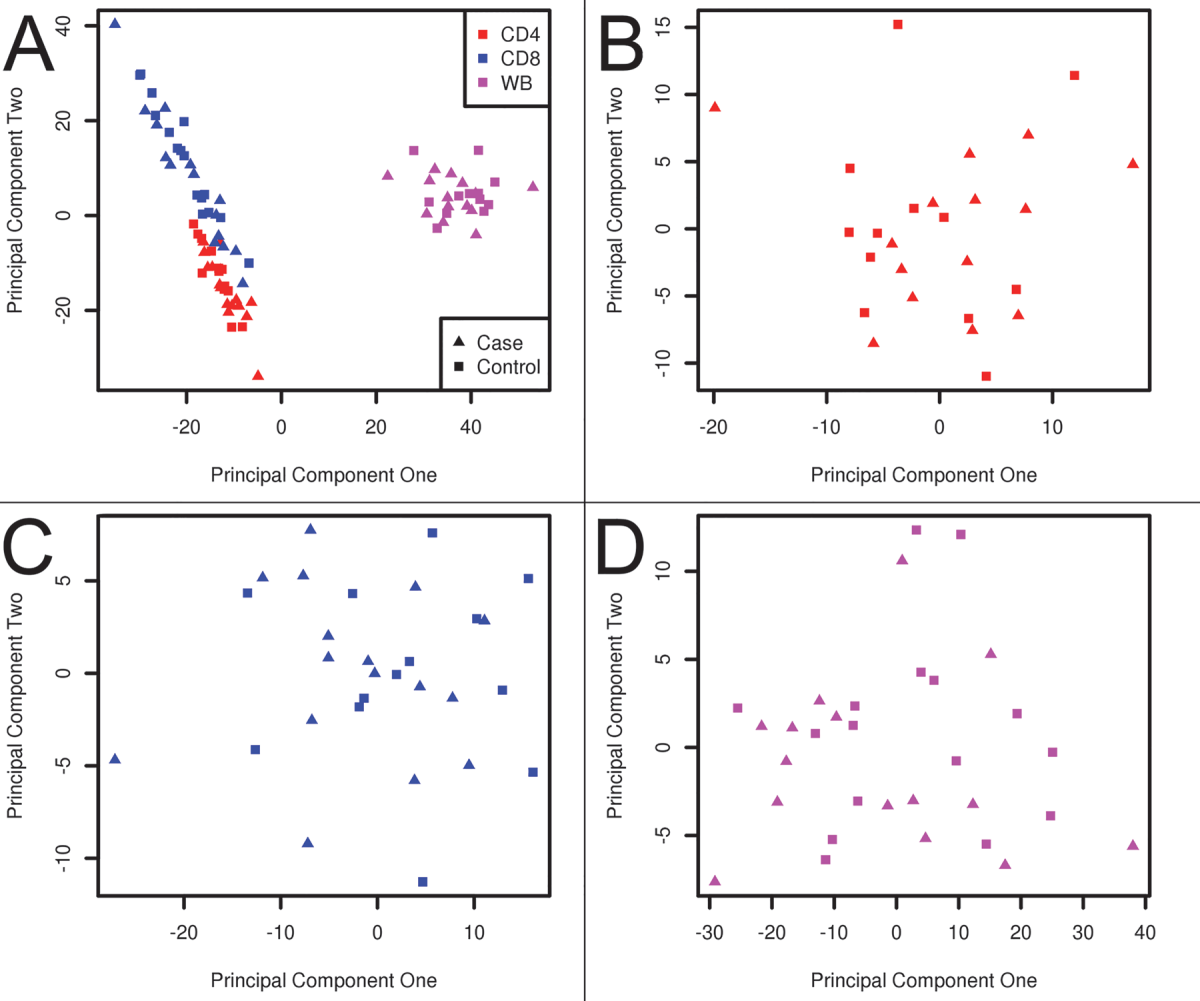
Principal component analyses. For samples in analyses a PCA was performed on overall methylation levels of CpG-sites that passed both quality controls and SNP filtering in (**A**) whole blood (Red), CD4+ T cells (Blue) and CD8+ T cells (Magenta) for all cases (squares) and controls (triangles). (**B**) PCA of DNA methylation data from whole blood only. (**C**) PCA of DNA methylation data from CD4+ T cells only. (**D**) PCA of DNA methylation data from CD8+ T cells only.

In order to prevent false positive signals due to genetic variation other than DNA methylation at probes, all probes that had an observed SNP in their target sequence (N = 60,106; see S1 Materials and Methods) in our data were removed before analysis[25] (S1B Fig.). To assess consistency of cell type specific methylation profiles, PCA of overall DNA methylation was applied (Fig. 1).

To account for cellular heterogeneity of WB, we adjusted for cell type distribution in our regression models. Sample-specific estimates of the cell type proportions were obtained by adapting the algorithm from Houseman *et al*.[26] using reference information on cell-specific methylation signatures[27]. Details on the procedures above are provided in S1 Materials and Methods.

### CpG-site differential methylation analysis

Two regression models were used in the analysis CpG-sites. In the first model we analyzed CD4+ T cell, CD8+ T cell or WB data separately, with ‘case-control’ status as a factor. Secondly, a two-way interaction model that utilized data from both CD4+ and CD8+ T cells was applied. In this model three factors were included; the ‘cell type’, the ‘group’ effect (case-control status), and an ‘interaction’ factor, which tested for statistical interaction between the cell type and case-control status. In case of statistical interaction between these two main factors, the DNA methylation directions are different between cell types across groups. To account for multiple testing we employed the Benjamini and Hochberg false discovery rate (FDR)[28]. CpG-sites with the lowest nominal p-values and at least 5% absolute difference in methylation[29] between MS patients and controls were examined. We examined the differences prioritized by lowest p-values to ensure the most consistently changing CpG-sites between MS cases and controls were considered. Fisher’s exact test was used to test for differences in distribution of all CpG-sites that reached nominal significance.

For the 5% of probes with the lowest p-values in the CD4+ and CD8+ T cell specific analyses, we determined whether support for any observed signal was present at neighboring CpG-sites. Our approach was based on the method described recently by Jaffe *et al*.[30]. Briefly, we defined a neighbor probe to be of interest if its p-value was also in the 5% of probes with lowest p-values for the respective cell type analyses, and the maximum distance between CpG-sites was not greater than 500 base pairs. If a neighbor hit was identified the algorithm then extended over the next 500 base pairs until no additional hits were present. We then grouped these individual CpG-sites into differentially methylated regions (DMRs). By permutation testing based on the area under the curve with respect to the test statistic we calculated p-values for these DMRs.

### Per-gene differential methylation analysis

The recently published list of MS-associated SNPs was used to define candidate genes (N = 148) for methylation differences given their putative role in the genetic predisposition to MS[4]. To account for multiple testing we also applied the FDR procedure[28]. CpG-sites were assigned to specific genes (N = 21,115) based on the provided Illumina manifest for the 450K. CpG-sites that mapped to multiple genes were included in analyses of all these genes. We used a permutation test based on the sum of the test statistics for each CpG-site within a gene.

## Results

### MS patient and control characteristics

Study characteristics are provided in Table 1. There were no significant differences between mean age or smoking status of MS patients compared to controls. All patients were diagnosed having RRMS, and the mean duration of disease was 8.8 years. The majority of patients had oligoclonal bands in their cerebrospinal fluid. All patients had modest EDSS and MSSS scores, and more than 10 typical MS lesions on cerebral MRI.

### Cell type specific DNA methylation profiles

PCA analysis of the DNA methylation profiles of CD4+ and CD8+ T cells as well as WB samples identified differences in the overall DNA methylation patterns between these cell types (Fig. 1A). Within each cell type, we did not observe clustering of the MS patients and controls, indicating that on a global level there are no large, consistent DNA methylation differences that distinguish individuals according to disease status. (Fig. 1B-D)

### Single CpG-site methylation analyses

In total 424,990 CpG-sites were considered after removal of CpG-sites with a low detection signal or SNPs in the probe sequence. Complete results from the per-CpG-site analysis using linear regression models are provided in S1 Table. We examined whether methylation differences observed in the T cell subsets were correlated with WB. Correlation of absolute mean differences from the WB data and either CD4+ and CD8+ T cell data was only moderate (respectively R^2^ = 0.51 and R^2^ = 0.56), whereas a higher correlation coefficient (R^2^ = 0.70) was observed for CD4+ and CD8+ T cells (S1C Fig.).

The 40 CpG-sites with the lowest nominal p-values and >5% absolute difference in methylation between MS patients and controls are listed in Table 2–4. For CD4+ and CD8+ T cells we also listed whether associated CpG-sites were in a DMR as defined above. All DMRs are provided in S2 Table. Two CpG-sites occurred in the top-40 for all three analyses, both were hypermethylated in MS patients compared to controls. The first of these two probes; cg05821046, is annotated at *TMEM48*, 622 base pairs upstream from the gene transcription start site. This CpG-site is located in a DMR of three CpG-sites, which was identified in both CD4+ and CD8+ T cell analyses (S2 Table, Chr1:54304846–54305115). *TMEM48* encodes a protein involved in the nuclear pore complex formation. The second probe; cg22560193, is located in the first exon of *APC2*, a gene predicted to be involved in microtubule and beta-catenin binding. Furthermore, several CpG-sites within *DNHD1* were also among the top 40 most differentially methylated in all three datasets. This gene encodes the dynein heavy chain domain like 1, which is a protein complex that is involved in microtubule movement. We note that after adjustment for multiple testing, none of these findings reached a genome-wide significance level (lowest adjusted p-value = 0.88, S1 Table).

**Table 2:** Top 40 results sorted by p-values from linear regression analysis models of DNA methylation in CD4+ T cells.

CD4+ T cells					
probeID^1^	Gene^2^	p-value^3^	Effectsize^4^	stdev^5^	p-value DMR (# probes in DMR)^6^
cg20585410	*DCX*	3.86E-05	-0.074	0.015	-
cg13988338	No gene	7.30E-05	-0.093	0.020	-
cg15552461	*RDH13*	9.58E-05	-0.069	0.015	-
cg01833234	***DNHD1***	1.49E-04	**0.145**	0.033	-
cg07937631	No gene	1.51E-04	**0.144**	0.033	-
cg24637308	***DNHD1***	1.63E-04	**0.108**	0.025	-
cg27419327	No gene	2.29E-04	-0.073	0.017	-
cg26477117	*TEKT5*	2.57E-04	-0.242	0.058	-
cg02336026	No gene	2.78E-04	-0.065	0.016	-
cg24431033	*TXNL1*	2.78E-04	**0.072**	0.017	5.5E-02 (3)
cg12543766	*MAGI2*	2.84E-04	-0.194	0.046	-
cg03700679	*TTC30B*	2.94E-04	**0.053**	0.013	-
cg06346838	***APC2***	3.88E-04	-0.062	0.015	-
**cg05821046**	*TMEM48*	4.03E-04	-0.065	0.016	7.0E-04 (3)
cg11213150	*ANGPTL2/RALGPS1*	4.06E-04	-0.054	0.013	-
cg08633479	*USP29*	4.11E-04	**0.066**	0.016	-
cg12243267	*USP29*	5.40E-04	**0.064**	0.016	-
cg06154311	*C20orf151*	5.68E-04	-0.075	0.019	-
cg27246129	*DLL1*	6.50E-04	-0.095	0.025	-
cg15627136	No gene	6.65E-04	-0.060	0.016	-
cg16288318	No gene	6.81E-04	-0.096	0.025	-
cg16259355	*DACH2*	7.49E-04	**0.064**	0.017	-
cg17332091	No gene	8.03E-04	-0.051	0.013	1.0E-03 (3)
cg23023970	*1INPP5A*	8.82E-04	-0.061	0.016	-
cg08682625	*LOC727677*	9.72E-04	**0.116**	0.031	-
cg04587084	No gene	1.03E-03	-0.070	0.019	-
cg10208301	***DNHD1***	1.08E-03	**0.129**	0.035	-
cg07733481	*SEMA5B*	1.15E-03	**0.148**	0.041	-
cg14667685	No gene	1.34E-03	-0.078	0.022	2.0E-03 (5)
**cg22560193**	*APC2*	1.39E-03	-0.089	0.025	-
cg14759977	*SUGT1L1*	1.44E-03	**0.051**	0.014	-
cg01413790	No gene	1.45E-03	-0.057	0.016	1.0E-03 (3)
cg06942183	*HOXB2*	1.51E-03	**0.068**	0.019	-
cg20954971	No gene	1.53E-03	-0.067	0.019	-
cg15015426	*OR10J5*	1.64E-03	-0.074	0.021	-
cg19285525	*RBMS1*	1.65E-03	-0.395	0.113	-
cg07019386	No gene	1.66E-03	-0.080	0.023	5.0E-02 (3)
cg17976205	*C20orf151*	1.74E-03	-0.052	0.015	-
cg22687569	No gene	1.79E-03	-0.120	0.035	-
cg00506935	*AEN*	1.87E-03	**0.062**	0.018	-

^1^Probe ID on 450K chip.

^2^Gene annotated to probe.

^3^p-value for specified probe in CD4+ T cells.

^4^Effect size of beta difference for specified probe. Positive values indicate hypomethylation of MS samples (i.e. controls DNA methylation higher than MS patients)

^5^Standard deviation for specified probe.

^6^Permutation-derived p-values for DMR in case the indicated probes is located in a DMR, in brackets we provided the number supportive CpG-sites in the respective DMRs.

Formatting legend

“Bold probeID” Specific probe occurs in all three data top-40 (see Tables 3, 4)

“*Bold Italic Gene*” Gene occurs in all three data top-40 (see Tables 3, 4)

”Bold Effectsize” Hypermethylation of probe in MS patients

Results shown are restricted to methylation differences of at least 5% (absolute beta difference). Full lists are provided in S1 Table.

**Table 3:** Top 40 results sorted by p-values from linear regression analysis models of DNA methylation in CD8+ T cells.

CD8+ T cells					
probeID^1^	Gene^2^	p-value^3^	Effectsize^4^	stdev^5^	p-value DMR (# probes in DMR)^6^
cg06346838	***APC2***	2.91E-06	-0.087	0.015	-
**cg22560193**	***APC2***	2.16E-05	-0.101	0.020	-
cg17332091	No gene	2.22E-05	-0.066	0.013	2.0E-05 (3)
cg13988338	No gene	4.61E-05	-0.093	0.019	-
cg10673318	No gene	5.39E-05	-0.062	0.013	-
cg19432993	*HOXA2*	6.94E-05	-0.066	0.014	1.3E-02 (5)
cg21995652	*HRNBP3*	1.43E-04	-0.055	0.012	-
cg24998110	*HEXDC*	1.47E-04	**0.060**	0.014	-
cg18772882	*NTRK3*	1.74E-04	-0.051	0.012	-
cg20971998	No gene	1.79E-04	-0.078	0.018	-
cg12580893	No gene	2.00E-04	-0.066	0.015	-
cg20585410	*DCX*	2.18E-04	-0.088	0.021	-
cg13560901	*TRIL*	2.86E-04	-0.072	0.017	-
cg20864214	*ARHGEF17*	2.95E-04	-0.090	0.022	-
cg07311615	*ESPNP*	2.95E-04	-0.068	0.016	2.0E-03 (2)
cg02225599	*HOXA2*	2.99E-04	-0.064	0.016	1.3E-02 (5)
cg09309261	*LHX5*	3.68E-04	-0.063	0.016	-
cg11902995	No gene	3.80E-04	-0.063	0.016	-
cg26477117	*TEKT5*	4.59E-04	-0.241	0.061	-
cg19225422	No gene	4.80E-04	-0.052	0.013	-
cg09213964	*LRRC43*	4.82E-04	-0.051	0.013	-
cg10173124	*CYP27C1*	5.21E-04	-0.052	0.013	-
**cg05821046**	*TMEM48*	5.36E-04	-0.097	0.025	2.2E-01 (3)
cg18782774	No gene	5.59E-04	-0.052	0.013	-
cg24938727	*HHATL*	6.39E-04	-0.061	0.016	-
cg00402910	*AMMECR1*	6.54E-04	-0.062	0.016	-
cg08065835	No gene	6.67E-04	-0.051	0.013	-
cg04764898	*C19orf45*	6.77E-04	-0.056	0.015	-
cg21686577	*SRRM3*	6.81E-04	-0.058	0.015	-
cg08387780	No gene	6.90E-04	-0.058	0.015	2.0E-05 (3)
cg01573321	*PSD3*	7.23E-04	-0.064	0.017	-
cg14531668	No gene	7.23E-04	-0.050	0.013	-
cg22970003	*PTPRN2*	7.63E-04	-0.073	0.019	-
cg14828182	*LOC654342*	7.63E-04	-0.062	0.016	-
cg20692922	No gene	7.65E-04	-0.078	0.021	-
cg16017089	*ARHGEF17*	7.79E-04	-0.059	0.016	-
cg24637308	***DNHD1***	7.84E-04	**0.086**	0.023	-
cg09307264	*KIF1C/INCA1*	8.08E-04	-0.052	0.014	-
cg05280762	*VSIG1*	8.08E-04	-0.054	0.014	-
cg25512439	*CNTN4*	9.38E-04	-0.060	0.016	-

^1^Probe ID on 450K chip.

^2^Gene annotated to probe.

^3^p-value for specified probe in CD8+ T cells.

^4^Effect size of beta difference for specified probe. Positive values indicate hypomethylation of MS samples (i.e. controls DNA methylation higher than MS patients)

^5^Standard deviation for specified probe.

^6^Permutation-derived p-values for DMR in case the indicated probes is located in a DMR, in brackets we provided the number supportive CpG-sites in the respective DMRs.

Formatting legend

“Bold probeID” Specific probe occurs in all three data top-40 (see Tables 2, 4)

“*Bold Italic Gene*” Gene occurs in all three data top-40 (see Tables 2, 4)

”Bold Effectsize” Hypermethylation of probe in MS patients

Results shown are restricted to methylation differences of at least 5% (absolute beta difference). Full lists are provided in S1 Table.

Results shown are restricted to methylation differences of at least 5% (absolute beta difference). Full lists are provided in S1 Table.

**Table 4:** Top 40 results sorted by p-values from linear regression analysis models of DNA methylation in whole blood samples.

Whole Blood				
probeID^1^	Gene^2^	p-value^3^	Effectsize^4^	stdev^5^
cg16259355	*DACH2*	6.95E-05	**0.109**	0.023
cg24493834	*LAMA2*	8.65E-05	**0.059**	0.012
cg23023844	*TTLL8*	1.16E-04	**0.138**	0.030
cg04903509	*GALNT9*	1.27E-04	**0.058**	0.013
cg20373036	*POU3F4*	2.25E-04	-0.059	0.014
cg00827196	No gene	3.63E-04	-0.051	0.012
cg16288318	*No gene*	3.98E-04	-0.147	**0.035**
cg00420742	NLRP12	5.07E-04	0.051	0.013
cg02336026	No gene	5.78E-04	-0.076	0.019
cg05052271	*PLS3*	5.87E-04	-0.070	0.018
cg01262952	*ANKRD1*	5.88E-04	**0.078**	0.020
cg02313554	No gene	7.35E-04	-0.138	0.036
cg13834112	No gene	7.86E-04	-0.051	0.013
cg25031670	No gene	8.17E-04	-0.084	0.022
cg25671428	*CLSTN2*	8.26E-04	-0.051	0.013
cg05141400	*MAGEB4*	8.60E-04	-0.086	0.023
cg01281231	No gene	8.85E-04	-0.054	0.014
cg25488749	No gene	8.92E-04	-0.052	0.014
**cg22560193**	***APC2***	9.08E-04	-0.091	0.024
cg27571374	No gene	9.31E-04	**0.137**	0.036
cg06076512	No gene	9.76E-04	**0.054**	0.014
cg11837293	No gene	1.02E-03	**0.058**	0.015
cg02851397	*PCDHA7*	1.06E-03	-0.081	0.022
cg17140469	No gene	1.08E-03	-0.066	0.018
cg20410114	No gene	1.08E-03	**0.053**	0.014
cg11336696	*TMEM27*	1.15E-03	-0.064	0.017
cg11185456	*DNHD1*	1.19E-03	**0.152**	0.041
cg06833709	*LGI1*	1.19E-03	-0.061	0.017
cg08243619	*PTCHD2*	1.19E-03	**0.081**	0.022
cg18618432	No gene	1.22E-03	-0.382	0.104
cg25523580	*MMD2*	1.24E-03	-0.089	0.024
cg24938727	*HHATL*	1.33E-03	-0.063	0.017
**cg05821046**	***TMEM48***	1.37E-03	-0.087	0.024
cg00399951	*NXPH1*	1.39E-03	-0.085	0.023
cg14336566	*TDRD9*	1.44E-03	**0.072**	0.020
cg23266594	*CDX1*	1.48E-03	-0.078	0.022
cg07465864	*YTHDC2*	1.51E-03	**0.066**	0.018
cg22351833	No gene	1.52E-03	-0.069	0.019
cg02778467	*RGPD1/PLGLB2*	1.58E-03	-0.091	0.025
cg25584862	No gene	1.62E-03	-0.052	0.015

^1^Probe ID on 450K chip.

^2^Gene annotated to probe.

^3^p-value for specified probe in whole blood.

^4^Effect size of beta difference for specified probe. Positive values indicate hypomethylation of MS samples (i.e. controls DNA methylation higher than MS patients)

^5^Standard deviation for specified probe.

Formatting legend

“Bold probeID” Specific probe occurs in all three data top-40 (see Tables 2, 3)

“*Bold Italic Gene*” Gene occurs in all three data top-40 (see Tables 2, 3)

”Bold Effectsize” Hypermethylation of probe in MS patients

Results shown are restricted to methylation differences of at least 5% (absolute beta difference). Full lists are provided in S1 Table.

Results shown are restricted to methylation differences of at least 5% (absolute beta difference). Full lists are provided in S1 Table.

**Fig 2:**
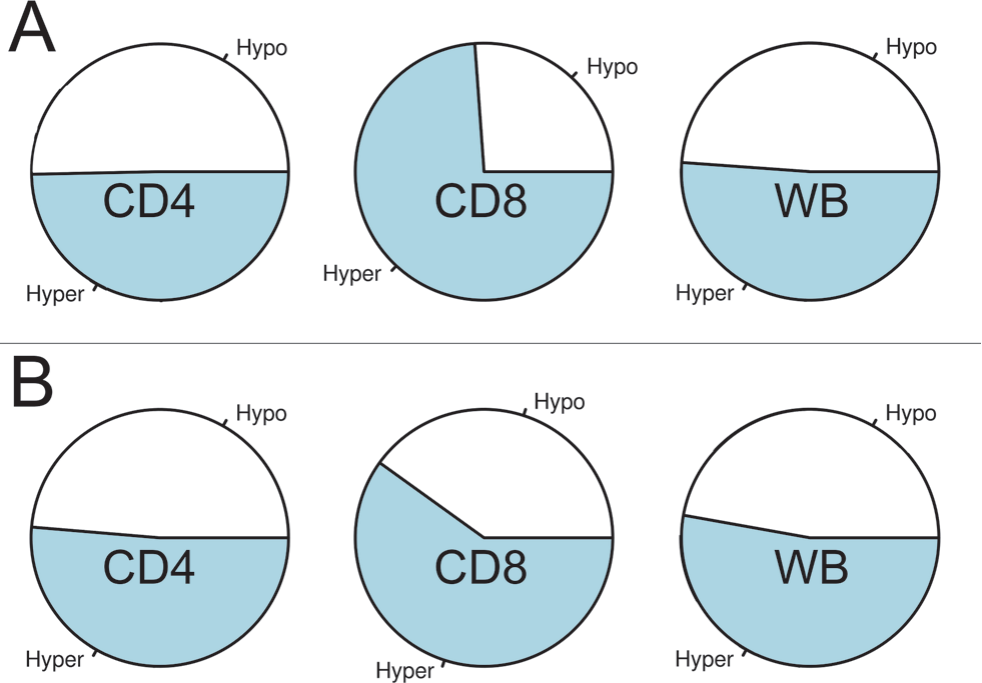
Pie charts of overall methylation levels for the three sample types. **A**. Pie-charts of DNA hyper- and hypomethylation for all CpG sites with p-values less then or equal to 0.05. **B**. Pie-charts of DNA hyper- and hypomethylation for all CpG-sites with p-values above 0.05. Abbreviations: Hypo – hypomethylation, Hyper – hypermethylation, CD4 – CD4+ T cell data, CD8 – CD8+ T cell data, WB – whole blood data.

Interestingly, for CD8+ T cells, 38 of the 40 most differentially methylated CpG-sites (95%) showed evidence for hypermethylation in MS patients when compared to controls. The *DNHD1* gene contained one of the only two hypomethylated CpG-sites in CD8+ T cells (Table 3). In contrast, a more balanced pattern was observed for both CD4+ T cells and WB; a much lower number of CpG-sites, 55% and 52.5%, respectively showed evidence for hypermethylation in MS patients, compared to controls (Table 2 and Table 4 respectively). When considering all CpG-sites with nominal p-values below 0.05 from the patient-control comparison, the proportion of hypermethylated CD8+ T cell CpG-sites in MS patients is significantly greater than hypomethylated CpG-sites (Fisher’s exact test p-value <0.01, Fig. 2A). DNA methylation of CpG-sites at different genomic features with respect to genes may provide additional insights in specific roles of the observed DNA hypermethylation in CD8+ T cells. When we considered genomic features for CpG-sites with p-values below 0.05, an overrepresentation of hypermethylated CpG-sites was slightly more frequent in 1,500 base pair regions upstream of the transcription start site (TSS-1500) and 1^st^ exon of genes (>76% hypermethylated sites) whereas the gene body and 3’-UTR show less evidence for hypermethylation; the lowest proportion (63%) of hypermethylated CpG-sites was observed in the 3’-UTR (data not shown). Furthermore, when we compared the more recently diagnosed patients (<7 years from diagnosis) with patients diagnosed earlier (>8 years from diagnosis) the more recently diagnosed patients showed a slightly higher proportion of DNA hypermethylation of their CD8+ T cells (proportion of hypermethylated sites 73% in recently diagnosed patients vs. 68% in the earlier diagnosed patients). We also examined CpG-sites for which patient-control comparisons did not yield p-values below 0.05, and the observation that CD8+ T cells are more likely to be hypermethylated remained, although less significant (Fig. 2B). For blood and CD4+ T cells, the distributions of hyper vs. hypomethylated CpG-sites were nearly identical (~50%) and not significantly different (Fig. 2A).

### Methylation differences between cell types

As expected, we observed large differences in DNA methylation profiles between CD4+ and CD8+ T cells. This was illustrated by the high total number of CpG-sites showing significant differences and the large differences of beta levels for these sites. Table 5 shows the 20 most significantly different CpG-sites among cell types, adjusted for disease status and possible interaction between disease status and cell type. Among these 20 CpG-sites none showed a case-control or interaction effect in the combined model. The CpG-sites showing the greatest differences among cell types had beta differences of up to 0.85, translating to an almost full switch of methylation status. Furthermore, the genes near or containing these CpG-sites have known roles in CD4+ T cell and CD8+ T cell regulation.

**Table 5:** Distinct differences between CD4+ and CD8+ T-cells observed in the ‘cell type’ term when applying a linear regression two-way interaction model including both the CD4+ and CD8+ T cell methylation data, including the terms ‘cell type’, ‘group’ (case-control status), and ‘interaction’ (case-control status x cell type).

				p-values^5^			
probeID^1^	Gene^2^	Effect size^3^	SD^4^	Cell Type	Cell Type BH corrected^6^	Group	Interaction
cg22505006	*ZBTB7B*	**0.849**	**0.008**	**1.61E-40**	**6.85E-35**	0.992	0.502
cg24955196	*ZBTB7B*	**0.724**	**0.007**	**3.27E-40**	**6.94E-35**	0.408	0.799
cg16871561	*SLC25A3*	**0.709**	**0.010**	**4.03E-35**	**5.71E-30**	0.918	0.290
cg25939861	*CD8A*	**-0.754**	**0.012**	**1.29E-34**	**1.37E-29**	0.314	0.824
cg06935361	*BRCA2*	**-0.669**	**0.011**	**1.97E-34**	**1.67E-29**	0.779	0.602
cg00219921	*CD8A*	**-0.764**	**0.013**	**3.41E-33**	**2.41E-28**	0.870	0.904
cg01782486	*ZBTB7B*	**0.656**	**0.012**	**6.61E-33**	**4.01E-28**	0.718	0.632
cg06449334	No gene	**-0.533**	**0.010**	**2.74E-32**	**1.46E-27**	0.299	0.557
cg25350872	*LOC154822*	**-0.530**	**0.010**	**4.35E-32**	**1.87E-27**	0.314	0.062
cg17343167	*N4BP3*	**-0.448**	**0.009**	**4.82E-32**	**1.87E-27**	0.503	0.467
cg24345747	*CD8A*	**-0.638**	**0.012**	**4.85E-32**	**1.87E-27**	0.370	0.396
cg19453665	*SERPINH1*	**-0.309**	**0.006**	**9.20E-32**	**3.16E-27**	0.392	0.891
cg03318654	*CD8A*	**-0.559**	**0.011**	**9.66E-32**	**3.16E-27**	0.408	0.947
cg03505866	*KIAA0247*	**0.437**	**0.009**	**1.14E-31**	**3.46E-27**	0.092	0.769
cg08934126	*CTNNBIP1*	**-0.309**	**0.006**	**1.42E-31**	**4.04E-27**	0.829	0.264
cg10837404	*DCP2*	**0.574**	**0.012**	**2.33E-31**	**6.19E-27**	0.460	0.357
cg26986871	No gene	**-0.565**	**0.011**	**3.33E-31**	**7.96E-27**	0.400	0.664
cg14477767	No gene	**0.716**	**0.015**	**3.37E-31**	**7.96E-27**	0.144	0.386
cg24462702	*CD40LG*	**0.378**	**0.008**	**4.38E-31**	**9.80E-27**	0.749	0.191
cg13798679	No gene	**-0.446**	**0.010**	**1.22E-30**	**2.59E-26**	0.326	0.835

^1^Probe ID on 450K chip.

^2^Gene annotated to probe.

^3^Effect size of beta difference for specified probe.

^4^standard deviation for specified probe.

^5^p-value for specified probe in respective models.

^6^Benjamini-Hochberg corrected p-values for factor "cell type”.

The top 20 highest-ranking probes sorted by p-values for differences of the ‘cell type’ term are listed, full lists are provided in S1 Table.

### MS candidate genes and exploratory per-gene analyses

Analysis of MS patients versus controls was performed at gene-level using a per-gene DNA methylation summary statistic for either CD4+ or CD8+ T cells. When considering CpG-sites annotated to genes of all established MS-associated SNPs[2], we observed no significant differences between MS patients and controls following correction for multiple testing (S3 Table). Similarly, no significant genes were observed when all genes covered by the 450K were taken into consideration (S3 Table).

## Discussion

Using a robust genome-wide DNA methylation profiling approach, we show no consistent large-effect DNA methylation differences for CD4+ T cells, CD8+ T cells or WB in a homogenous collection of MS patients and controls. However, while nominally significant methylation differences were small, CD8+ T cell DNA from MS patients showed strong evidence for hypermethylation at a large number of these CpG-sites. Furthermore, we confirmed large-effect, genome-wide significant DNA methylation differences between CD4+ T cells and CD8+ T cells, underscoring the importance of separating different immune cell subpopulations in DNA methylation studies. Although none of the MS patient-control DNA methylation analyses reached genome-wide significance, we observed two CpG-sites with low p-values for all the three different sample types. We cannot exclude the possibility that genetic variation other than DNA methylation could underlie such consistent results; however, given the dense genotype information we obtained, and lack of a known SNP in the probe sequences[31], our evidence strongly suggests a consistent DNA methylation difference between MS patients and controls is present. The first CpG-site, measured by probe cg05821046 resides in a DMR including two additional probes for both CD4+ and CD8+ T cells (Tables 2 and 3). The lead CpG-site is localized upstream of *TMEM48*, a gene encoding the nuclear pore complex protein NDC1. Little is known about this protein and its potential role in MS. The second consistent CpG-site difference was measured by probe cg22560193 and is annotated to the last exon of gene *APC2*. This CpG-site is not located in a DMR when considering the CpG-sites covered by the 450K. *APC2* encodes the protein adenomatosis polyposis coli 2, which is mainly expressed in neuronal tissue. The relevance of increased DNA methylation of CpG-sites within this gene in immune cells from MS patients is unclear.

Remarkably, the CD8+ T cells of MS patients showed a predominantly higher level of DNA methylation compared to controls for those CpG-sites with the lowest p-values. Since the canonical role of DNA methylation at gene promoters is gene silencing and we observed a slightly higher percentage of hypermethylated sites in these promoter regions, it is possible that gene silencing in circulating CD8+ T cells of MS patients may be present. Whether this observation persists in a larger study warrants further investigation.

After correcting for multiple testing, we did not find significant evidence for association between per-gene DNA methylation within specifically candidate genes[2], or when all genes on the 450K were considered. It is important to note that the 450K covers only a portion of the CpG-sites present in the human genome. Although the array is gene centric and largely encompasses potential regulatory regions, it is possible that MS-associated DNA methylation differences exist outside the CpG-sites covered by this array. Given the complex disease aetiology in MS, at individual patient level, changes in DNA methylation may still contribute to disease-risk.

While the sample size in this study is modest, we had at least 80% power to detect beta-value differences of 0.05 and larger, assuming per-CpG-site median standard deviations (S1D Fig.). Thus, for half of the CpG-sites, the power to detect a beta difference over 0.05 was over 80%. Therefore, our study had power to detect large-effect, consistent methylation differences between MS patients and controls. The observed hypermethylation in CD8+ T cells has small effect sizes and none of the CpG-sites reached genome-wide significance individually. A PCA of genome-wide SNP data[20] allowed us to verify Nordic ancestry and excluded systematic genetic differences between patients and controls in the study. Methylation levels for specific loci might change with age and differ between gender[32]; therefore, only female MS patients and female, age matched controls were included in this study. The clinical data show these MS patients are representative of an average MS population with a relative benign disease course. Importantly, since medication may influence DNA methylation[33], the MS patients selected for this study had never used immune-modulatory drugs at time of sampling or received steroids for at least three months prior to inclusion. Furthermore, since tobacco smoke is a known driver of methylation differences in peripheral blood cells[34], we also performed an analysis including smoking status as a covariate; however, this did not substantially change the results (data not shown).

A recent study by Graves *et al*. reported significant DNA methylation changes within CD4+ T cells of the MHC region in MS patients using the 450K[35]. In our study, we noted 18 of 19 (95%) of these CpG- sites within the MHC were compromised by the presence of at least one SNP in the probe sequence[25]. For the remaining CpG-site in the MHC, we did not observe a nominally significant difference. Furthermore, a SNP was present in the probes for 8 of 55 associated CpG-sites outside the MHC region. None of the remaining 47 non-MHC CpG-sites reached significance in our study. Therefore, we could not confirm the findings reported by Graves *et al*.[35]. Notably, our sample was smaller, though more clinically homogeneous with respect gender and disease course. The high number of excluded CpG-sites due to the presence of a SNP in the probe sequence underscores the need for genotype-based filtering of chip-based DNA methylation data. Alternatively, probes that might contain SNPs[25] can be identified by utilizing publicly available data[36].

Our results are in agreement with Baranzini *et al*., who applied reduced bisulphite sequencing covering over 2 million CpG-sites, and showed no consistent large-scale methylation differences in MS discordant twins and siblings[15]. The reported switch of methylation from 20% to 80% for CpG-sites close to the *TMEM1* or *PEX14* genes between discordant twins could not be examined, since these CpG-sites are not included on the 450K.

Temporality must be considered in DNA methylation studies. It remains possible that MS patient DNA methylation profiles deviated from healthy controls at disease onset and are no longer detectable. When we consider the more recently diagnosed patients these showed a high proportion of DNA hypermethylation of their CD8+ T cells. The patients that were diagnosed earlier also show a profound DNA hypermethylation, though the proportion is slightly lower as compared to the recently diagnosed patients. We cannot exclude the possibility that the disease process in itself affects DNA methylation. This possibility must be investigated in a longitudinal cohort of MS patients.

For use as possible biomarkers of MS in the clinic, characteristic DNA methylation profiles should preferably be identified in easily obtainable WB. After correction of the WB methylation profiles in our dataset according to Houseman *et al*.[26], the correlation coefficients of WB compared to T cells remained moderate (S1C Fig.). Therefore, we cannot conclude that WB will reliably reflect disease relevant changes in T cells, however additional studies on the biomarker value of DNA methylation profiles derived from WB are warranted.

In conclusion, this is the first study of genome-wide DNA methylation profiles derived from WB, CD4+ and CD8+ T cells, in homogenous, untreated female MS patients and matched controls. We identified strong evidence for DNA hypermethylation in CD8+ T cells of MS patients. The significant methylation differences observed between CD4+ T cells, CD8+ T cells and WB underscore the importance of considering cell-based profiles. Further, more sophisticated algorithms for correction of individual variability in cell proportions are needed, if DNA methylation profiles from WB are to be used reliably. Based on available power, we excluded large-scale individual and per-gene DNA methylation differences between patients and controls, for CpG-sites tested here. In particular, large DNA methylation differences for CpG-sites within 148 established MS candidate genes tested in the current study do not explain missing heritability. Larger studies of homogenous MS patients and controls are warranted to further elucidate the impact of smaller DNA methylation changes that may be important in MS pathogenesis.

## Supporting Information

S1 Fig Supplementary figures S1A-D. A. Principal component analysis (PCA) of MS patients and controls used in the methylation analyses (respectively triangles and squares in color). The principal components for samples in current study were plotted against those derived from an earlier large GWAS study of Norwegian MS patients and controls. Results showthe samples in the DNA methylation study cluster within the Nordic population. **B**. SNPs in methylation probes influence reported beta values; example of a SNP located in the sensing probe sequence of CpG-site cg21139150 resulting correlation between reported beta-values and sample genotype. **C**. Scatterplot of –log(p-values) of the per-probe patient-control analysis for CD8+ T cell test statistics against CD4+ T cell test statistics, resulting in a correlation coefficient R^2^ = 0.70. **D**. Post-hoc power calculations for increasing quintiles of observed probe variance.
(TIF)

S1 Materials and Methods Detailed materials and methods for procedures briefly described in manuscript. (DOCX)

S1 Table Per-probe analyses details. (ZIP)

S2 Table All DMR analyses details. (XLSX)

S3 Table Per-gene analyses details. (XLSX)
