# Supplementary figures and images for: Genome-Wide DNA Methylation Profiles Indicate CD8+ T Cell Hypermethylation in Multiple Sclerosis
Source: PLoS One. 2015 Mar 3;10(3):e0117403. doi: 10.1371/journal.pone.0117403 (PMC4348521; doi:10.1371/journal.pone.0117403)

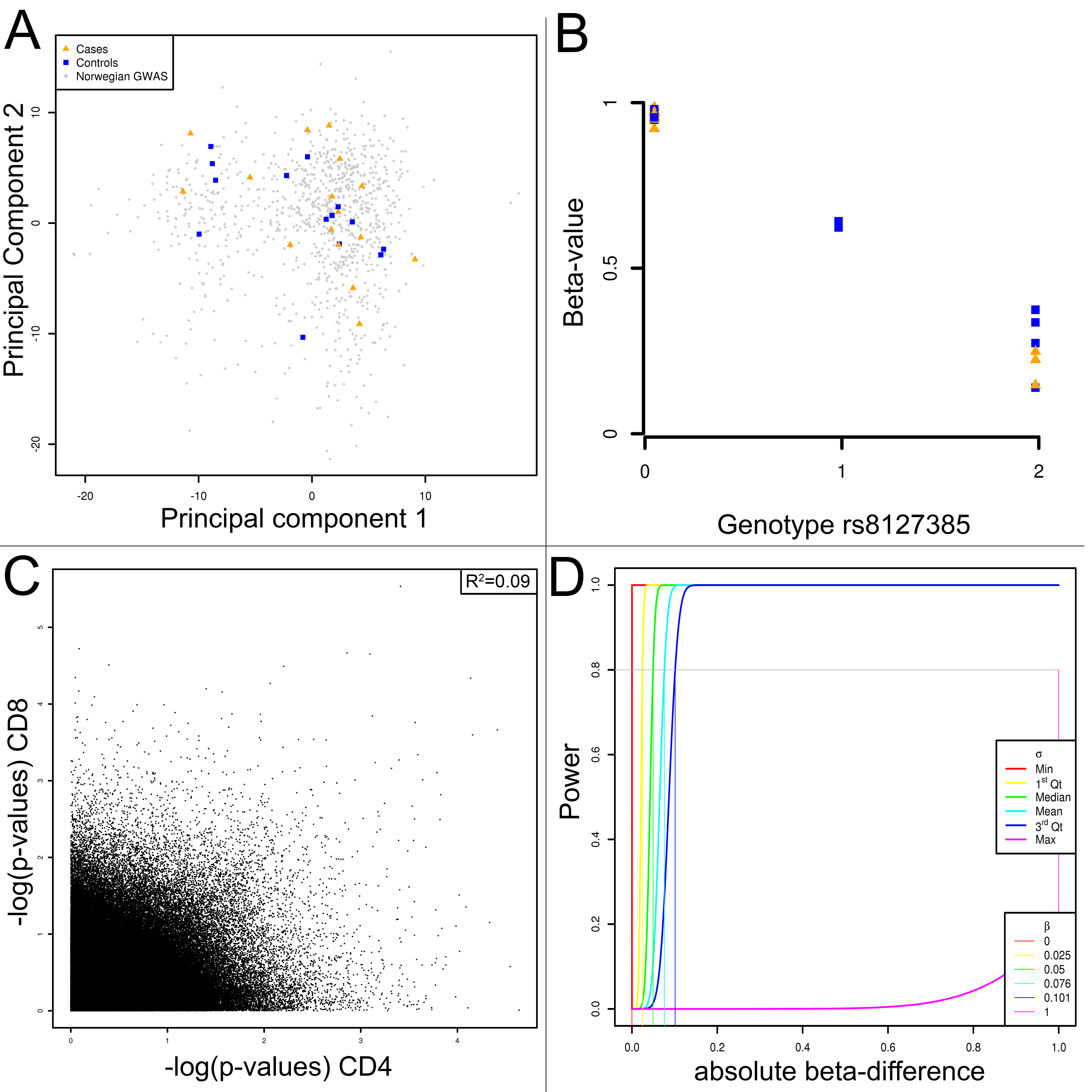

Supplement: S1 Fig — A. Principal component analysis (PCA) of MS patients and controls used in the methylation analyses (respectively triangles and squares in color). The principal components for samples in current study were plotted against those derived from an earlier large GWAS study of Norwegian MS patients and controls. Results showthe samples in the DNA methylation study cluster within the Nordic population. B. SNPs in methylation probes influence reported beta values; example of a SNP located in the sensing probe sequence of CpG-site cg21139150 resulting correlation between reported beta-values and sample genotype. C. Scatterplot of –log(p-values) of the per-probe patient-control analysis for CD8+ T cell test statistics against CD4+ T cell test statistics, resulting in a correlation coefficient R2 = 0.70. D. Post-hoc power calculations for increasing quintiles of observed probe variance. (TIF) [file pone.0117403.s001.tif]
