# Supplementary material for: Genome-Wide DNA Methylation Profiles Indicate CD8+ T Cell Hypermethylation in Multiple Sclerosis
Source: PLoS One. 2015 Mar 3;10(3):e0117403. doi: 10.1371/journal.pone.0117403 (PMC4348521; doi:10.1371/journal.pone.0117403)
Supplement: S1 Materials and Methods — (DOCX) [file pone.0117403.s002.docx]

**Supplementary Materials and Methods**

**Blood collection**

EDTA coated vacuum tubes (Greiner Bio-One, Frickenhausen, Germany) were used to collect 64 ml of whole blood, which was transferred to a 100 ml culture flask containing 1 ml of 100 mM EDTA solution (Life Technologies, Paisley, UK). The total volume of the blood was adjusted to 140 ml using RPMI culture medium (Life Technologies, Paisley, UK). Four equal volumes of diluted blood were carefully pipetted onto 15 ml of lymphoprep (Sigma-Aldrich, Oslo, Norway) in 50 ml tubes (Greiner Bio-One, Frickenhausen, Germany) and the peripheral blood mononuclear cells (PBMCs) were separated from the other blood constituents by centrifugation at 800g for 30 minutes. The PBMCs were washed three times in ice-cold PBS before suspension in 2 ml ice cold PBS.

**Cell separation**

PBMCs were counted using nucleocount NC-100 (ChemoMetec A/S, Denmark) and pelleted, followed by suspension in a volume of 80 μl MACS buffer per 10 million cells. Per 10 million cells, 20 μl of magnetic anti-CD8+ beads (Miltenyi Biotec, Lund, Sweden) was mixed with the cells and incubated for 15 minutes at 4**°**C, washed with MACS buffer and resuspended in 500 μl of MACS buffer. The autoMACS cell separator was used to separate positive and negative cell fractions (positive selection) and the positive fraction was counted and kept on ice. The CD8+ negative fraction was centrifuged for 10 minutes at 300g and the supernatant removed. The pellet was suspended in 30 μl per 10 million cells MACS buffer and 10 μl per 10 million cells of biotin labeled CD4+ negative antibody cocktail was added and incubated for 10 minutes at 4**°**C. To this suspension 10 μl per million cells and 10 μl per million cells anti-biotin magnetic beads were added and incubated at 4**°**C for 15 minutes. The cells were washed in MACS buffer before resuspension in 500 μl of MACS buffer. The autoMACS cell separator (Miltenyi Biotec, Lund, Sweden) was used to separate positive and negative cell fractions (negative selection) and the negative fraction was counted and kept on ice, whereas the positive fraction was discarded. For each separated cell type, aliquots of 1-3 million cells were stored at -20**°**C.

**Flow Cytometry assessment of cell fraction purities**

During collecting for the majority of samples flow cytometry was performed as detailed below. All CD4+ and CD8+ T cell fractions demonstrated 95% or greater purity. Cells were labelled with FITC-conjugated mouse anti-human CD4 (Clone RTF-4g), mouse anti-human CD8 (clone RTF-8) or mouse IgG1 isotype control (clone 15H6) (all from Southern Biotech), and cell purity was assessed by flow cytometry on FACS Calibur (BD Biosciences) and data analysed by Cell Quest Pro (BD Biosciences). During collecting for the majority of samples flow cytometry was performed and all CD4+ and CD8+ T cell fractions demonstrated 95% or greater purity.

**DNA isolation and QC**

DNA was isolated from the cell pellets using Qiamp DNA mini kit (Qiagen, Sweden) by adding 200 μl of lysis buffer to the thawed cell pellets before following the instructions as provided by the manufacturer. DNA was quantified using the nanodrop spectrophotometer (Thermo Scientific, Wilmington, DE 19810 USA) and samples with 260/280 values below 1.7 were subjected to additional purification by precipitation and dissolving in extraction buffer.

**Imputation of genotypes against the 1,000 genomes reference panel**

Genotypes of the Illumina 660 Quad array were pre-phased using phase-it. Phased data was then imputed using impute2[22], applying the 1,000 genomes central European reference panel. We applied a 90% information threshold for calling genotypes of imputed SNPs.

**Illumina 450K Methylation Array measurements**

750 ng of the DNA was used as input for the bisulfite conversion using the Zymo EZ-96DNA Methylation Kit (Catalog #D5004) Deep-Well Format. Then 4 μl of the bisulfite converted DNA was used as input for the Illumina Infinium HD Methylation Assay according to the manufacturer’s protocol. Samples are transferred to Illumina's Infinium HumanMethylation450K DNA Analysis BeadChip before scanning on the Illumina HiScan.

Once scanning was completed, the data was uploaded into GenomeStudio for preliminary analysis and QC. Target success rate was determined. The detection p-value is the 1-p computed from the background model characterizing the chance that the target sequence was distinguishable from the negative controls. CpG-sites with more than 20% missing values were excluded from the analysis (CD4+ T cells N=72; CD8+ T cells N=72; whole blood (WB) N=67; overlap for all categories N=49). Sample replicates and Jurkat cell DNA control replicates are checked to ensure an r^2^ value of greater than 0.99. No irregularities were observed in the on-array internal controls provided by Illumina.

**SNP assessment for probes**

By overlaying the probe genomic locations as provided by Illumina with the imputed and genotyped per-sample SNP map, we identified all probes that contain at least one observed or imputed polymorph site in their sequences. These probes (N=60,106) were removed from analyses in all samples to prevent false methylation readouts (Figure S1B).

**Correlation of WB, CD4+ T and CD8+ T cell DNA profiles**

For each CpG-site in the WB, CD4+ and CD8+ T cells data, absolute differences in beta values were calculated. Correlations of these absolute differences were assessed for the WB to either CD4+ or CD8+ T cell data, and for CD4+ T cell data to CD8+ T cell data. The latter comparison is illustrated by Figure S1C.
